# Stress, coping, and quality of life in the United States during the COVID-19 pandemic

**DOI:** 10.1371/journal.pone.0277741

**Published:** 2023-05-10

**Authors:** Fathima Wakeel, Jacelyn Hannah, Leah Gorfinkel

**Affiliations:** Department of Community and Population Health, College of Health, Lehigh University, Bethlehem, Pennsylvania, United States of America; University of Colorado Denver, UNITED STATES

## Abstract

While research has widely explored stress, coping, and quality of life (QOL) individually and the potential links between them, a critical dearth exists in the literature regarding these constructs in the context of the COVID-19 pandemic. Our study aims to identify the salient stressors experienced, describe the coping strategies used, and examine the relationships between stressors, coping, and QOL among individuals during the pandemic. Data are from a sample of 1,004 respondents who completed an online survey. Key measures included stressful life events (SLEs), coping strategies, and the physical and psychological health domains of QOL. Staged multivariate linear regression analyses examined the relationships between SLEs and the two QOL domains, controlling for sociodemographic and pre-existing health conditions and testing for the effects of coping strategies on these relationships. The most common SLEs experienced during the pandemic were a decrease in financial status, personal injury or illness, and change in living conditions. Problem-focused coping (β = 0.42, σ = 0.13, *p* < 0.001 for physical QOL; β = 0.57, σ = 0.12, *p* < 0.001 for psychological QOL) and emotion-focused coping (β = 0.86, σ = 0.13, *p* < 0.001 for psychological QOL) were significantly related to higher levels of QOL, whereas avoidant coping (β = –0.93, σ = 0.13, *p* < 0.001 for physical QOL; β = -1.33, σ = 0.12, p < 0.001 for psychological QOL) was associated with lower QOL. Avoidant coping partially mediated the relationships between experiencing SLEs and lower physical and psychological QOL. Our study informs clinical interventions to help individuals adopt healthy behaviors to effectively manage stressors, especially large-scale, stressful events like the pandemic. Our findings also call for public health and clinical interventions to address the long-term impacts of the most prevalent stressors experienced during the pandemic among vulnerable groups.

## Introduction

The COVID-19 pandemic led to a prolonged period of stress due to its extensive adverse impacts, including increased mortality and morbidity, substantial economic challenges, heightened levels of uncertainty, and social isolation. Exposure to chronic stressors can significantly impact one’s physical and psychological health directly through the neuroendocrine system (i.e., recurring activation of neuroendocrine responses and consequent increases in cholesterol, blood sugar, triglycerides, and blood pressure) or immune system (i.e., impairment of the immune system and the resulting risk of infection) pathways, as well as indirectly through unhealthy behaviors such as poor diet, smoking, substance misuse, and risky sexual behaviors [1–9]. Experiencing stressful life events (SLEs) has also been significantly associated with reduced quality of life (QOL) in a wide range of vulnerable populations, including racial and ethnic minorities [10], elderly persons [11–13], chronically ill patients [14–16], and children [17,18]. Specifically, SLEs have been found to be inversely associated with physical QOL [13,16] and psychological QOL [16].

The World Health Organization [19] defines quality of life (QOL) as “individuals’ perception of their position in life in the context of the culture and value systems in which they live, and in relation to their goals, expectations, standards and concerns” (p.1405). Some research has indicated that individuals experienced lower QOL during the pandemic. For example, stressors, such as loss of income, personal health effects, social isolation, and COVID-19 diagnosis, were negatively correlated with QOL during the pandemic [20]. Further, a study [21] in Germany found that the pandemic did not affect the QOL of individuals equally, with women, job seekers, and younger people reporting a significantly lower QOL. This study also indicated an overall decline in reported physical and psychological QOL during the pandemic [21].

Individuals cope with stressors in various ways. Three coping strategies widely investigated in the literature include problem-focused coping, emotion-focused focusing, and avoidant coping. Each of these strategies entails different methods for dealing with SLEs. Problem-focused coping involves stress-reducing tactics such as problem-solving, obtaining instrumental support, and planning [22,23]. Emotion-focused coping strategies include the use of emotional support, humor, religion, and positive reframing [22,23]. Avoidant coping involves behaviors such as substance use, distractions, and behavioral disengagement [24]. The literature has demonstrated that problem-focused coping is the most effective in stressful situations because it entails taking control of the stressor and using proactive methods to address it [23]. On the other hand, emotion-focused coping has been shown to be most effective when the stressor, such as a death of a loved one, is outside of one’s control [23,25]. Avoidant coping may be the least effective and most harmful because it not only does not remove the stressor but also likely worsens existing stress, anxiety, and depression [26–28].

There is limited information about the role of coping strategies in the relationships between SLEs and QOL. In a study by Merluzzi et al. [15], disengagement/denial coping, which is a form of avoidant coping, mediated the associations between SLEs and emotion QOL and physical/functional QOL among cancer patients. Inconsistent findings exist regarding the relationships between SLEs, coping strategies, and other outcomes such as depression, anxiety, life satisfaction, loneliness, and distress. In a 20-year longitudinal study among high-school students in Canada, Pelekanakis et al. [29] found that problem-focused coping moderated, but did not mediate, the association between SLEs and depressive symptoms in that this association was lower among individuals who employed problem-focused coping. Conversely, emotion-focused coping both mediated and moderated the relationship between SLEs and depressive symptoms, such that SLEs were positively associated with emotion-focused coping, which was then positively associated with depressive symptomatology; additionally, the relationship between SLEs and depressive symptoms was stronger among those who used emotion-focused coping. The study did not demonstrate a mediating or moderating effect for avoidant coping. However, avoidant coping was found to moderate the relationships between SLEs and depression, anxiety, and life satisfaction among college students during the pandemic [30]. Conversely, avoidant coping has been shown to mediate the associations between SLEs and cancer-related distress [31], as well as depression, anxiety, and loneliness [32].

Further, though several studies have captured how frequently various coping strategies were used during the COVID-19 pandemic, there is limited research on the relationships between coping strategies and QOL during this time period. A large-scale study [33] in the United Kingdom found that emotion-focused coping strategies were more likely to be used when individuals experienced financial stressors (i.e., their or their partner’s loss of employment or inability to work, decrease in household income) or worries about contracting or becoming severely sick from COVID-19 and that both problem-focused coping and avoidant coping strategies were likely to be employed when respondents reported adverse financial events as well as worries about finances, basic needs, or contracting COVID-19. In terms of potential links between coping and QOL during the pandemic, research [34] has shown that for patients hospitalized with COVID-19, the use of problem-focused coping mechanisms had a direct and positive correlation with their QOL. Additionally, Quiroga-Garza et al. [35] found that problem-focused and emotion-focused coping were marginally, but significantly, correlated with well-being. Further, Shamblaw et al. [28] found that approach coping, which entails more proactive aspects of problem-focused and emotion-focused coping (e.g., planning, positive reframing, and use of emotional support), was associated with higher QOL, whereas avoidant coping was related to significantly lower QOL during the pandemic.

While researchers have widely explored stress, coping, and QOL individually, as well as potential links between them, there is a critical dearth in the literature regarding these constructs in the context of the COVID-19 pandemic. As the pandemic had far-reaching and multidimensional health impacts on the population, it provided a unique opportunity to investigate how individuals cope with stressors during a relatively brief period of time and how different coping strategies may have differential impacts on individuals’ QOL. Our study uses a nationwide sample of over 1000 U.S. adults to 1) Identify the salient stressors reported by individuals during the pandemic; 2) Describe the types of coping strategies used by individuals during the pandemic; and 3) Examine the relationships between stressors, coping, and QOL among individuals during the pandemic. Based on findings in the extant coping literature, we hypothesize that problem-focused coping and emotion-focused coping during the pandemic were positively associated with QOL. Further, we predict that experiencing SLEs was positively related to the use of avoidant coping, which, in turn, was negatively associated with QOL.

## Materials and methods

### Sample and procedures

Data for this study are from a nationwide sample of 1,004 respondents who completed our 25-30-minute online survey on *Prolific*, a web-based survey recruitment platform, in August 2021. Respondents were provided with the informed consent statement and were able to access the survey only after indicating their consent and that they met the eligibility criteria (i.e., residing in the U.S., being 18 years of age or older) for the study. This study was approved by the Lehigh University Institutional Review Board.

Survey questions were grouped into three main categories: 1) Well-being topics, including QOL, social relationship factors, and stress and coping; 2) Information topics, including sources of health information, telehealth services, and consumption behavior; and 3) Science and vaccines topics, including vaccine intentions and behaviors, attitudes and beliefs about scientific and medical research, and political and religious preferences. To reduce the potential respondent burden, respondents were randomly assigned two out of the three categories of questions. Therefore, though 1,500 respondents completed the overall survey, only 1,004 individuals completed the questions relevant to this study.

### Measures

#### Quality of life

QOL was measured using the World Health Organization Quality of Life Abbreviated Version (WHOQOL-BREF) instrument [36]. This 26-item measure asks the respondent to indicate how often or how completely certain experiences relating to general QOL and various dimensions of QOL, including physical health, psychological health, social relationships, and environmental health, occurred in the past four weeks using 5-point Likert scale responses (1 = “not at all” or never”; 5 = “an extreme amount,” “completely,” or “always”). Scores were summed for each QOL dimension, with higher scores indicating higher levels of QOL. Scores for each QOL dimension were then rescaled to range from 0–100, per the instrument’s scoring instructions. In this analysis, we focused on the dimensions of physical health (7 items) and psychological health (6 items), as these dimensions measured perceptions regarding more internal experiences of health, as opposed to experiences with external factors such as social relationships and environmental situations. The physical and psychological health measures (α = 0.82 for both) had good internal consistency (Table 1).

**Table 1:** Descriptive statistics and correlations between study measures.

	Cronbach’s alpha	Mean	SD	Range	1	2	3	4	5	6
1. Stressful life events	N/A	1.7	2.2	0–18	1.00	0.21*	0.20*	0.31*	-0.23*	-0.18*
2. Problem-focused coping	0.78	13.8	5.4	0–24		1.00	0.57*	0.26*	0.07*	0.12*
3. Emotion-focused coping	0.67	11.1	5.1	0–24			1.00	0.33*	0.03	0.14*
4. Avoidant coping	0.75	9.1	4.9	0–24				1.00	-0.24*	-0.33*
5. Quality of life (physical health)	0.82	70.4	19.7	0–100					1.00	0.61*
6. Quality of life (psychological health)	0.82	64.1	21.3	0–100						1.00

*p<0.05.

#### Stressors

Stressors were measured using a modified version of the Holmes and Rahe Rating Scale [37], in which we asked respondents if they experienced any of the following 21 stressful life events (SLEs) during the pandemic: Death of spouse/partner, child, close family member, or close friend; personal injury or illness; domestic violence in the home; injury or illness in a child or other family member(s); divorce; separation from partner; imprisonment; loss of employment; loss of employment of spouse/partner; loss of educational opportunity; pregnancy; pregnancy of spouse/partner; childbirth; childbirth by spouse/partner; decrease in financial status; need to cut the size meals or skip meals because there wasn’t enough money for food; homelessness; foreclosure of mortgage or loan; eviction; change in living conditions; and increase in the frequency of arguments at home or work. Respondents were asked to mark all that applied, and we used the following categories of responses in our analyses: 0, 1, 2, 3, and 4+ SLEs. We also examined the individual effects of each SLE in our multivariate regression models. SLEs were not operationalized as a composite score because over half of the respondents reported not experiencing any SLEs during the pandemic, and the distribution of the composite measure was wide. Further, due to the skew in the SLE count distribution (i.e., 352 individuals experiencing no SLEs, 236 experiencing one SLE, 161 experiencing two SLEs, 113 experiencing three SLEs, 65 experiencing four SLEs, and 77 experiencing 5+ SLEs), we decided to truncate the operationalization of the SLE measure at 4+ SLEs in order to ensure a more even distribution among the SLE categories (i.e., 35%, 24%, 16%, 11%, and 14% of sample reporting 0, 1, 2, 3, and 4+ SLEs, respectively). Other research [38–40] examining SLEs has utilized this approach.

#### Coping

Coping style was measured using a 16-item version of Carver’s Brief COPE measure [24]. Dias et al. [41] grouped the Brief COPE items into three coping styles: problem-focused coping, emotion-focused coping, and avoidant coping. In our survey, individuals were asked to indicate, using 5-point Likert scale responses (1 = “I haven’t been doing this a lot”; 5 = “I have been doing this a lot”), how they coped with stressors over the past year. Problem-focused coping included four items relating to active coping and the use of informational support. Emotion-focused coping included six items pertaining to emotional support, humor, and religion. Avoidant coping had six items relating to self-distraction, substance use, and behavioral disengagement. Scores for each coping strategy were summed, with higher scores reflecting greater use of that strategy. We rescaled the problem-focused coping scores to be out of 24 points to compare our findings more easily regarding different coping styles. The problem-focused, emotion-focused, and avoidant coping measures had acceptable internal consistencies with Cronbach alphas of 0.78, 0.67, and 0.75, respectively (Table 1).

#### Sociodemographic variables

The following sociodemographic variables were included in the analyses: race/ethnicity, gender identity, annual household income, age, and marital status.

Race/ethnicity categories included American Indian or Alaska Native; Asian; Black or African American; Hispanic, Latino or Spanish Origin; Middle Eastern or North African; Native Hawaiian or Other Pacific Islander; White; other; and prefer not to say. Due to their low frequencies in our sample, we categorized American Indian or Alaska Native, Middle Eastern or North African, Native Hawaiian or Other Pacific Islander, and Other as “Other Race/ethnicity” for the analyses.

We included the following gender identity categories in the survey: cisgender male; cisgender female; transgender male; transgender female; non-binary/gender non-conforming; do not identify as female, male, or transgender; and prefer not to say. Our analysis grouped transgender, non-binary/gender non-conforming, and non-identifying individuals as “other gender identity” due to their low frequencies in the sample.

Annual household income was operationalized as the following categories: Less than $25,000; $25,000-$34,999; $35,000- $49,999; $50,000-$74,999; $75,000-$99,999; $100,000-$149,999; $150,000-$199,999; greater than or equal to $200,000; and prefer not to say. The analysis grouped individuals with annual household incomes of $150,000 to $199,999 and $200,000 or more due to their smaller frequencies.

Age was a continuous variable that was provided as an open-ended response.

Marital status categories included: single/never married; married; not married, but in a relationship and living with your partner; not married, but in a relationship and not living with your partner; separated; divorced; widowed; and other.

#### Pre-existing health conditions

Pre-existing health conditions that were included in the analyses were having a chronic physical condition, mental health condition, or disability.

Chronic physical health condition was operationalized as a dichotomous variable (i.e., any versus none) indicating whether the respondent reported being diagnosed with at least one of the following chronic illnesses: Multiple sclerosis; high blood pressure; COPD; diabetes; heart disease (heart failure, aFib, etc.); cancer; autoimmune (Psoriatic disease, Crohn’s/Ulcerative Colitis, etc.); asthma; rheumatoid arthritis; and other.

Mental health condition was a dichotomous variable (any versus none); conditions included the following: Mood disorder (e.g., depression, bipolar disorder, etc.); anxiety disorder (e.g., obsessive-compulsive disorder, panic disorder, phobias, etc.); eating disorder (e.g., anorexia, bulimia, etc.); Post-traumatic Stress Disorder; and other.

Disability was operationalized as a dichotomous variable (any versus none) indicating if the respondent reported being diagnosed with any of the following disabilities: Sensory impairment (vision or hearing); mobility impairment; learning disability (e.g., ADHD, dyslexia); and other.

### Analytical approach

**Fig 1:**
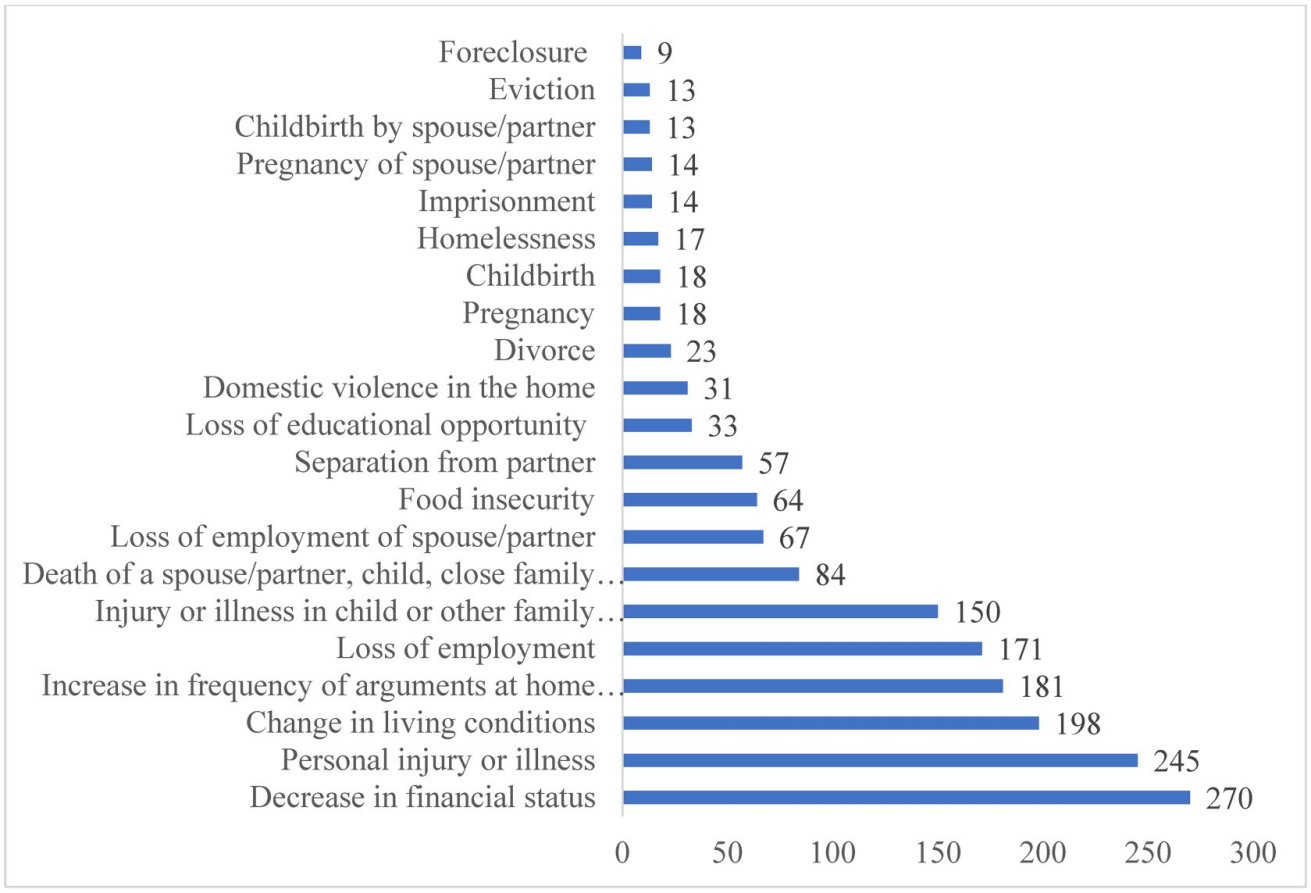
Frequency of stressful life events during the COVID-19 pandemic. Fig 1 demonstrates the frequency of stressful life events experienced by respondents during the COVID-19 pandemic.

The analyses were conducted using RStudio, version 4.1.2 [42]. Frequency distributions of SLEs were obtained (Fig 1). The internal consistency (i.e., Cronbach’s alphas) of the study measures, as well as correlations (i.e., Pearson product-moment correlation coefficients) between the measures, were calculated (Table 1). Kruskal-Wallis tests were conducted to compare the means of QOL, SLEs, and coping strategy measures by sociodemographic and health-related characteristics (Table 2). Staged multivariate linear regression analyses were then conducted to examine the relationships between each of the two QOL dimensions (i.e., psychological health and physical health) and SLEs, controlling for sociodemographic and pre-existing health conditions and testing for the effects of coping strategies on these relationships. The following covariates were included in the six models: 1) Model 1: SLE categories (i.e., 4+ events, three events, two events, one event); 2) Model 2: Model 1 covariates, sociodemographic variables, and pre-existing health conditions; 3) Model 3: Model 2 covariates, problem-focused coping, and avoidant coping; 4) Model 4: Model 2 covariates and emotion-focused coping, and avoidant coping; 5) Model 5: Model 2 covariates, problem-focused coping, and emotion-focused coping; and 6) Model 6: Model 5 covariates and avoidant coping (Tables 3 and 4). Overall, missing values (i.e., three in total) in the data wereminimal and imputed using the average of existing responses.

**Table 2:** Means of quality of life, stressful life events, and coping strategies scores by sociodemographic and health characteristics.

	Quality of Life Physical Health (Range 0–100)		Quality of Life Psychological Health (Range 0–100)		Stressful Life Events (Range 0–21)		Problem-focused coping (Range 0–24)		Emotion-focused coping (Range 0–24)		Avoidant coping (Range 0–24)	
	Mean (S.D.)	*p*	Mean (S.D.)	*p*	Mean (S.D.)	*p*	Mean (S.D.)	*p*	Mean (S.D.)	*p*	Mean (S.D.)	*p*
n = 1,004	70.44 (19.67)		64.13 (21.26)		1.68 (2.16)		13.79 (5.35)		11.07 (5.11)		9.13 (4.89)	
**Sociodemographic variables (%)**												
Race/ethnicity		0.87		**<0.001**		0.19		0.15		**0.02**		0.29
Asian (6.0%, n = 60)	70.8 (16.5)		60.3 (20.7)		1.5 (1.7)		13.4 (4.7)		10.1 (5.1)		9.6 (4.8)	
Black or African American (11.7%, n = 117)	73.3 (18.7)		66.3 (24.9)		1.4 (1.4)		14.8 (6.0)		12.4 (5.2)		9.4 (5.1)	
Hispanic, Latino, or Spanish Origin (3.6%, n = 36)	70.8 (19.8)		53.8 (22.4)		1.9 (1.6)		14.0 (4.5)		12.0 (4.1)		9.6 (3.2)	
Multiracial (4.5%, n = 45)	70.9 (18.1)		58.2 (21.8)		2.1 (2.1)		14.9 (4.3)		11.3 (5.2)		10.2 (4.1)	
White (73.1%, n = 734))	70.1 (20.0)	ref	65.2 (20.4)	ref	1.7 (2.3)	ref	13.6 (5.4)	ref	10.9 (5.1)	ref	8.9 (5.0)	ref
Other/Prefer not to say (1.2%, n = 12)	54.2 (20.6)		51.4 (21.3)		2.0 (1.6)		13.6 (3.1)		10.5 (5.4)		9.4 (3.5)	
Annual household income		**0.03**		**<0.001**		0.13		**0.02**		**<0.001**		**<0.001**
Less than $25,000 (16.0%, n = 163)	64.8 (22.5)		57.7 (22.2)		1.7 (1.8)		13.6 (6.0)		10.9 (5.5)		9.4 (5.0)	
$25,000 to $34,999 (8.4%, n = 84)	63.4 (23.4)		59.7 (21.7)		1.9 (1.9)		12.8 (5.6)		9.4 (4.9)		7.6 (4.8)	
$35,000 to $49,999 (14.5%, n = 146)	69.2 (18.9)		59.0 (21.8)		1.5 (1.7)		13.2 (4.8)		10.9 (4.8)		9.0 (4.3)	
$50,000 to $74,999 (17.1%, n = 172)	71.2 (20.5)		66.3 (19.4)		1.7 (1.7)		14.1 (5.5)		11.2 (5.3)		8.9 (4.8)	
$75,000 to $99,999 (14.0%, n = 141)	75.7 (17.5)		67.2 (19.5)		1.5 (2.1)		13.4 (5.1)		10.8 (4.8)		8.4 (3.6)	
$100,000 to $149,999 (16.0%, n = 161)	72.9 (14.4)		70.3 (19.4)		2.1 (3.5)		14.8 (5.2)		12.6 (5.0)		10.7 (5.4)	
$150,000 or more (11.1%, n = 111)	75.6 (17.1)	ref	69.1 (21.4)	ref	1.3 (1.4)	ref	13.7 (5.4)	ref	10.8 (5.0)	ref	9.0 (5.0)	ref
Prefer not to say (2.6%, n = 26)	64.4 (20.0)		56.6 (22.7)		2.2 (2.1)		14.9 (3.5)		10.7 (4.6)		9.2 (3.4)	
Gender identity		0.11		**<0.001**		0.09		0.17		0.38		0.17
Cisgender female (47.0%, n = 472)	70.2 (19.7)		61.3 (20.5)		1.6 (1.7)		14.1 (5.1)		11.3 (5.1)		8.9 (4.5)	
Cisgender male (43.7%, n = 439)	71.3 (18.7)	ref	67.7 (21.5)	ref	1.7 (2.5)	ref	13.4 (5.6)	ref	10.7 (5.1)	ref	9.2 (5.2)	ref
Other gender identity (2.0%, n = 20)	64.5 (20.3)		49.2 (25.4)		3.1 (4.0)		14.9 (6.1)		12.3 (4.4)		11.1 (4.5)	
Prefer not to say (7.3%, n = 73)	68.6 (24.5)		65.6 (19.5)		1.4 (1.6)		13.9 (5.5)		11.1 (5.2)		9.6 (5.3)	
Marital status		0.06		**<0.001**		0.20		0.12		**0.002**		**0.008**
Divorced (9.6%, n = 96)	66.6 (24.5)		61.6 (21.8)		1.5 (1.8)		13.2 (5.5)		9.6 (5.2)		8.1 (4.3)	
Married (46.7%, n = 469)	72.1 (19.0)		70.4 (19.4)		1.7 (2.6)		13.8 (5.6)		11.5 (5.3)		9.0 (5.2)	
Not married, but in a relationship and living together (7.7%, n = 77)	66.9 (17.9)		57.6 (18.9)		1.8 (2.0)		14.9 (5.0)		11.6 (4.6)		10.3 (5.0)	
Not married, but in a relationship and not living together (5.5%, n = 55)	71.9 (17.2)		57.0 (20.0)		1.8 (1.8)		15.4 (4.5)		12.2 (4.2)		10.5 (4.4)	
Separated (0.6%, n = 6)	55.4 (27.4)		56.3 (31.5)		1.3 (1.8)		12.8 (1.6)		8.2 (5.2)		7.3 (4.1)	
Single/never married (27.0%, n = 271)	70.1 (18.9)		56.9 (21.3)		1.7 (1.6)		13.5 (5.2)		10.6 (4.9)		9.3 (4.4)	
Widowed (2.2%, n = 22)	67.7 (26.6)		72.7 (21.5)		1.2 (1.1)		12.8 (6.2)		10.5 (5.7)		7.1 (5.9)	
Unknown or prefer not to say (0.8%, n = 8)	74.1 (15.9)		64.1 (23.4)		2.4 (2.3)		14.4 (4.1)		10.3 (4.7)		8.4 (5.9)	
Age ^a^	0.01 (-0.05, 0.07)	0.71	0.27 (0.22, 0.33)	**<0.001**	-0.21 (-0.26, -0.15)	**<0.001**	-0.21 (-0.27, -0.15)	**<0.001**	-0.25 (-0.30, -0.19)	**<0.001**	-0.36 (-0.41, -0.30)	**<0.001**
**Pre-existing health conditions (%)**												
Any chronic physical health condition		**<0.001**		0.52		0.08		**<0.001**		**0.005**		**<0.001**
Yes (45.4%, n = 456)	65.6 (20.8)		63.6 (20.8)		1.9 (2.5)		13.2 (5.2)		10.5 (5.1)		8.6 (4.9)	
No (54.6%, n = 548)	74.5 (17.7)	ref	64.6 (21.7)	ref	1.5 (1.8)	ref	14.3 (5.4)	ref	11.5 (5.1)	ref	9.6 (4.8)	ref
Any mental health condition		**<0.001**		**<0.001**		**<0.001**		0.10		0.10		**<0.001**
Yes (34.7%, n = 348)	63.7 (20.7)		54.4 (21.3)		2.0 (2.0)		14.2 (5.1)		11.4 (4.8)		10.1 (4.7)	
No (65.3%, n = 656)	74.0 (18.1)	ref	69.3 (19.4)	ref	1.5 (2.2)	ref	13.6 (5.5)	ref	10.9 (5.3)	ref	8.6 (4.9)	ref
Any disability		**<0.001**		**<0.001**		**<0.001**		0.16		0.94		0.39
Yes (20.4%, n = 205)	60.5 (22.3)		58.8 (22.6)		2.3 (2.4)		13.6 (5.0)		11.0 (5.0)		9.4 (5.0)	
No (79.6%, n = 799)	73.0 (18.1)	ref	65.5 (20.7)	ref	1.5 (2.1)	ref	13.8 (5.4)	ref	11.1 (5.1)	ref	9.1 (4.9)	ref

ref = reference group; Bolded p-values are statistically significant;

^a^Pearson correlation coefficients (with 95% confidence intervals) for age are provided instead of means.

**Table 3:** Relationships between number of stressful life events and quality of life (physical health).

Significant covariates^a^	Model 1		Model 2		Model 3		Model 4		Model 5		Model 6	
	β (SE)	*p*	β (SE)	*p*	β (SE)	*p*	β (SE)	*p*	β (SE)	*p*	β (SE)	*p*
1 stressful life event	-1.95 (1.59)	0.22	-1.00 (1.51)	0.51	-1.02 (1.47)	0.49	-0.67 (1.47)	0.65	-1.43 (1.51)	0.34	-1.01 (1.47)	0.49
2 stressful life events	-8.71 (1.79)	<0.001	-5.99 (1.75)	<0.001	5.01 (1.71)	0.004	-4.62 (1.72)	0.007	-6.37 (1.75)	<0.001	-4.94 (1.72)	0.004
3 stressful life events	-13.35 (2.04)	<0.001	-9.17 (1.99)	<0.001	-8.03 (1.95)	<0.001	-7.55 (1.96)	<0.001	-9.75 (1.99)	<0.001	-7.98 (1.96)	<0.001
4+ stressful life events	-14.39 (1.87)	<0.001	-10.14 (1.87)	<0.001	-8.72 (1.86)	<0.001	-8.00 (1.86)	<0.001	-11.05 (1.88)	<0.001	-8.67 (1.86)	<0.001
“Other” race/ethnicity			-11.06 (5.23)	0.03	-10.62 (5.07)	0.04	-10.58 (5.10)	0.04	-11.22 (5.20)	0.03	-10.64 (5.07)	0.04
Income of $25,000-$34,999			-6.02 (2.56)	0.02	-6.83 (2.49)	0.006	-6.97 (2.51)	0.006	-5.80 (2.55)	0.02	-6.84 (2.49)	0.006
Chronic physical health condition			-6.90 (1.25)	<0.001	-6.76 (1.22)	<0.001	-6.95 (1.22)	<0.001	-6.70 (1.25)	<0.001	-6.77 (1.22)	<0.001
Mental health condition			-7.08 (1.25)	<0.001	-6.22 (1.21)	<0.001	-6.19 (1.22)	<0.001	-7.13 (1.24)	<0.001	-6.20 (1.21)	<0.001
Disability			-7.78 (1.45)	<0.001	-7.55 (1.41)	<0.001	-7.76 (1.42)	<0.001	-7.71 (1.45)	<0.001	-7.61 (1.41)	<0.001
Problem-focused coping					0.49 (0.11)	<0.001			0.39 (0.13)	0.003	0.42 (0.13)	<0.001
Emotion-focused coping							0.39 (0.12)	0.001	-0.03 (0.14)		0.15 (0.14)	
Avoidant coping					-0.90 (0.13)	<0.001	-0.91 (0.13)	0.001			-0.93 (0.13)	<0.001

^a^Only statistically significant coefficients, with the exception of the stressful life event categories, are reported in this table.

**Table 4:** Relationships between number of stressful life events and quality of life (psychological health).

Significant covariates^a^	Model 1		Model 2		Model 3		Model 4		Model 5		Model 6	
	β (SE)	*p*	β (SE)	*p*	β (SE)	*p*	β (SE)	*p*	β (SE)	*p*	β (SE)	*p*
1 stressful life event	-5.11 (1.70)	0.003	-3.17 (1.55)*	0.04	-3.59 (1.45)	0.01	-3.06 (1.44)	0.03	-4.11 (1.50)	0.006	-3.52 (1.42)	0.01
2 stressful life events	-15.20 (1.92)	<0.001	-10.21 (1.81)	<0.001	-9.25 (1.70)	<0.001	-8.44 (1.68)	<0.001	-10.91 (1.75)	<0.001	-8.87 (1.66)	<0.001
3 stressful life events	-15.35 (2.18)	<0.001	-8.97 (2.05)	<0.001	-8.00 (1.93)	<0.001	-7.14 (1.91)	<0.001	-10.25 (1.99)	<0.001	-7.72 (1.90)	<0.001
4+ stressful life events	-14.98 (2.01)	<0.001	-7.93 (1.92)	<0.001	-6.90 (1.84)	<0.001	-5.69 (1.81)	0.002	-10.00 (1.87)	<0.001	-6.60 (1.80)	<0.001
Black race/ethnicity			3.89 (1.90)	0.04								
Income of $25,000-$34,999			-4.63 (2.64)	0.08	-5.49 (2.47)	0.03	-5.72 (2.44)	0.02	-4.06 (2.55)	0.11	-5.54 (2.42)	0.02
Income of $35,000-$49,999			-4.53 (2.24)	0.04	-4.12 (2.09)	0.049	-5.28 (2.07)	0.01	-4.87 (2.16)	0.02	-4.88 (2.05)	0.02
Cisgender female			-3.34 (1.27)	0.009	-4.63 (1.18)	<0.001	-4.96 (1.17)	<0.001	-4.21 (1.22)	<0.001	-5.12 (1.16)	<0.001
“Other” gender identity			-9.27 (4.24)	0.03	-9.69 (3.95)	0.01	-9.87 (3.91)	0.01	-10.09 (4.09)	0.01	-10.02 (3.87)	0.01
Divorced			-5.63 (2.17)	0.01	-5.54 (2.03)	0.006	-4.01 (2.01)	0.046	-4.84 (2.10)	0.02	-4.41 (1.99)	0.03
In a relationship and cohabiting			-6.27 (2.36)	0.008	-6.23 (2.20)	0.005	-4.70 (2.18)	0.03	-6.09 (2.28)	0.008	-5.25 (2.16)	0.02
In a relationship and not cohabiting			-6.92 (2.81)	0.01	-7.34 (2.61)	0.005	-5.98 (2.59)	0.02	-6.40 (2.71)	0.02	-6.41 (2.56)	0.01
Single/never married			-7.10 (1.62)	<0.001	-6.95 (1.51)	<0.001	-5.66 (1.51)	<0.001	-5.35 (1.58)	<0.001	-5.74 (1.49)	<0.001
Age			0.23 (0.05)	<0.001	0.18 (0.04)	<0.001	0.21 (0.04)	<0.001	0.30 (0.05)	<0.001	0.21 (0.04)	<0.001
Chronic physical health condition			-2.59 (1.29)	0.04	-2.23 (1.20)	0.06	2.56 (1.19)	0.03	-2.21 (1.25)	0.08	-2.32 (1.18)	0.049
Mental health condition			-10.87 (1.28)	<0.001	-9.81 (1.20)	<0.001	-9.67 (1.19)	<0.001	-11.01 (1.24)	<0.001	-9.69 (1.18)	<0.001
Problem-focused coping					0.98 (0.11)	<0.001			0.52 (0.13)	<0.001	0.57 (0.12)	<0.001
Emotion-focused coping							1.18 (0.11)	<0.001	0.60 (0.14)	<0.001	0.86 (0.13)	<0.001
Avoidant coping					-1.17 (0.12)	<0.001	-1.30 (0.13)	<0.001			-1.33 (0.12)	<0.001

^a^Only statistically significant coefficients are reported in this table.

## Results

### Description of sample

The sample was predominantly White (73%, n = 734), followed by Black (12%, n = 117), Asian (6.0%, n = 60), Latinx (4%, n = 36), and multiracial individuals (5%, n = 45) (Table 2). When compared to the racial and ethnic distribution in the U.S. [43], our sample had similar distributions of the racial categories, but only 4% of our sample reported being Hispanic (compared to 19% of the U.S. population). The sample was evenly spread out through the annual household income categories; while our distribution of annual household income was fairly similar to that in the U.S., substantially fewer respondents (11%, n = 111) reported having an income of greater than $150,000 when compared to the U.S. (20%) [44]. The sample was relatively equally distributed by cisgender male and female, with only 2% (n = 20) of respondents in the “other” category (transgender, non-binary, or does not identify as male, female, or transgender). Almost 50% (n = 469) of the sample was married, and 27% (n = 271) were single/never married. The age distribution of respondents ranged from 18 to 82 years old, and the mean age was 44. Further, 45% (n = 456) of the sample reported having a chronic physical health condition, 35% (n = 348) reported having a mental health condition, and 20% (n = 205) reported having a disability.

The mean count of SLEs was 1.6, with a large spread within the sample ranging from zero to eighteen events (Table 2). The three most prevalent SLEs reported in the sample were a decrease in financial status, followed by personal injury or illness and a change in living conditions (Fig 1). Further, on average, the respondents reported higher levels of problem-focused coping, followed by emotion-focused coping and avoidant coping (Table 2).

### Bivariate analysis

Table 1 illustrates the descriptive statistics of the study measures as well as the correlations between measures. Bivariate analyses indicate that SLEs were positively associated with all three coping strategies (.21, p<0.001 for problem-focused coping; .20, p<0.001 for emotion-focused coping; and .31, p<0.001 for avoidant coping) and negatively associated with both QOL dimensions (-.23, p<0.001 for physical health and -.18, p<0.001 for psychological health). Problem-focused coping was weakly, positively correlated with both QOL dimensions (.07, p = 0.028 for physical health and .12, p<0.001 for psychological health), emotion-focused coping was weakly, positively associated with QOL psychological health (.14, p<0.001), and avoidant coping was negatively correlated with both QOL dimensions (-.24, p<0.001 for physical health and -.33, p<0.001 for psychological health).

Significant unadjusted racial and ethnic differences in QOL psychological health and emotion-focused coping were found (Table 2). Latinx individuals reported the lowest QOL psychological health, whereas Asian individuals reported the lowest emotion-focused coping scores. Black individuals reported the highest scores for both measures. Post-hoc analysis (i.e., Dunn test with Bonferroni adjustment) revealed that there were significant differences in QOL psychological health between Black and Latinx individuals (p = 0.023; data not shown in table). Further, there were significant differences by annual household income in QOL physical health, QOL psychological health, and all three coping strategies. Reported QOL generally increased with income levels. Based on post-hoc analysis, in general, there were significant differences in both QOL domains when comparing individuals with the lowest incomes (i.e., less than $25,000 and $25,000-$49,000) to those with the highest incomes (i.e., $150,000 or higher, $100,000-$149000). There were also significant differences in psychological health among more proximal income levels; for instance, those with incomes of $50,000-$74,999 had significantly different QOL psychological health scores compared to those with incomes less than $25,000 (p = 0.009). No consistently identifiable pattern emerged with coping strategies; individuals who had an income between $25,000 and $35,000 reported the lowest scores for all three types of coping, and individuals who had an income of $100,000-$150,000 generally reported the highest scores for all three types of coping. Additionally, the only significant differences in the gender identity variable were observed in QOL psychological health, with those who identified as “other” gender identity reporting the lowest QOL and cisgender males reporting the highest QOL.

There were significant differences by marital status for QOL psychological health, emotion-focused coping, and avoidant coping. Separated individuals generally reported the lowest scores across these measures, and those in a relationship but not cohabiting reported the highest emotion-focused and avoidant coping scores. Married and widowed individuals reported the highest QOL psychological health (Table 2). Specifically, post-hoc analysis indicated that married individuals reported significantly higher QOL psychological health compared to those who were divorced (p = 0.005), not married and living with a partner (p<0.001), not married and not living with their partner (p<0.001) and single/never married (p<0.001); further, widowed individuals reported higher QOL psychological health compared to individuals who were not married and living with a partner (p = 0.016) or were single/never married (p = 0.038). Further, those with a chronic health condition, mental health condition, or disability reported significantly lower QOL physical health. Individuals with a mental health condition or disability also reported significantly lower QOL psychological health and a higher number of SLEs. Those with a chronic health condition reported significantly lower scores for the three types of coping, and those with a mental health condition reported higher avoidant coping scores.

### Multivariate analysis

Staged multivariate linear regression analyses examined the relationships between QOL (the physical and psychological health dimensions), SLEs, and the three coping strategies. Table 3 displays the statistically significant covariates in each of the models for which the QOL physical health dimension was the outcome variable. Based on the final model (model 6), experiencing a greater number of SLEs was associated with increasingly lower QOL physical health (β = -4.94, σ = 1.72, p = 0.004 for 2 SLEs; β = -7.98, σ = 1.96, p<0.001 for 3 SLEs; and β = -8.67, σ = 1.86, p < 0.001 for 4+ SLEs. After adjusting for all other covariates, problem-focused coping was positively associated with QOL physical health (β = 0.42, σ = 0.13, *p* < 0.001), whereas avoidant coping was negatively associated with this outcome (β = -0.93, σ = 0.13, *p* < 0.001). Other significant covariates that were associated with significantly lower QOL physical health included “other” race/ethnicity (β = -10.64, σ = 5.07, *p* = 0.04), having an annual household income between $25,000-$34,999 (β = -6.84, σ = 2.49, *p* = 0.006), and having a chronic physical health condition (β = -6.77, σ = 1.22, *p* < 0.001), mental health condition (β = -6.20, σ = 1.21, *p* < 0.001), or disability (β = -7.61, σ = 1.41, *p* < 0.001).

**Fig 2:**
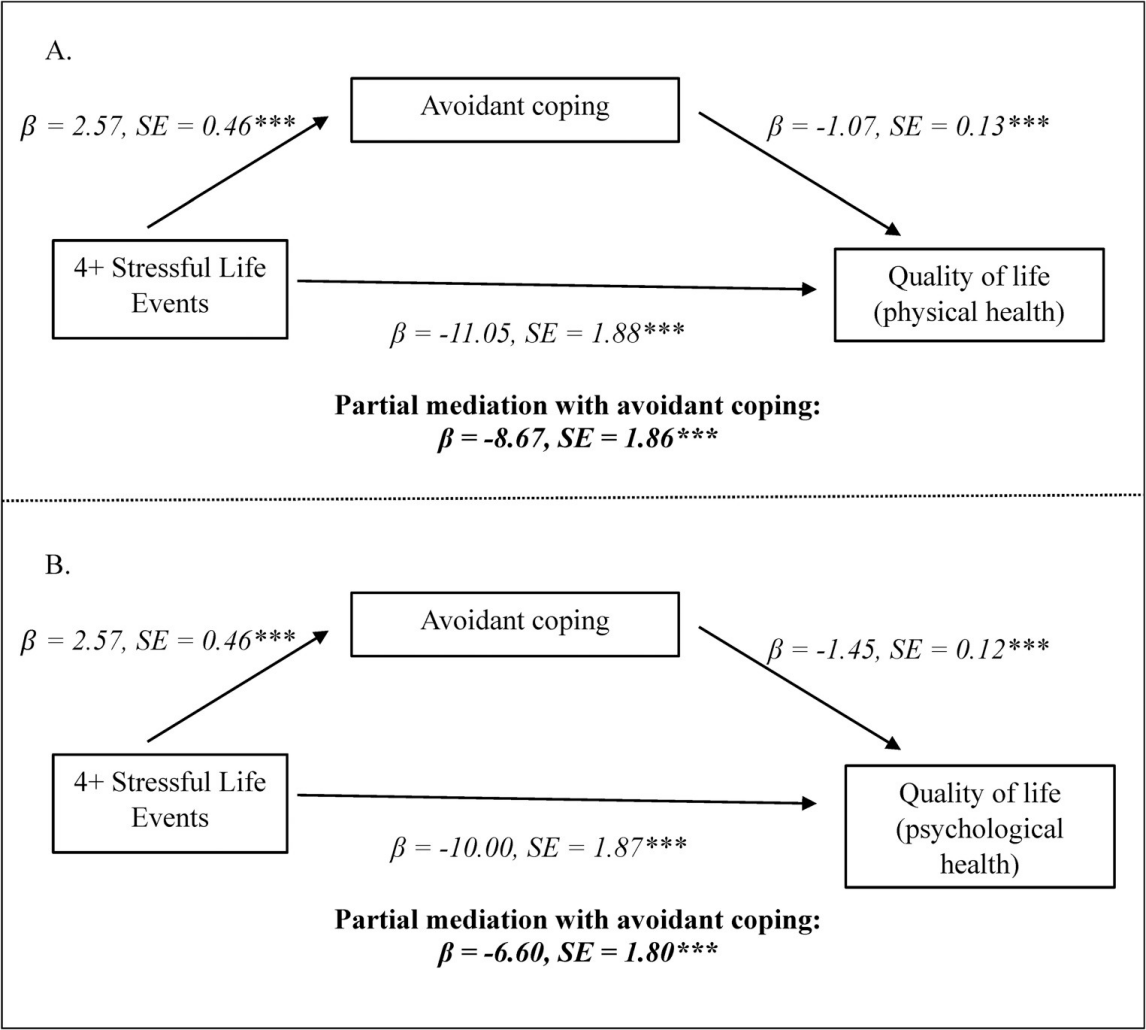
Mediation models relating stressful life events, avoidant coping, and quality of life. Fig 2A demonstrates that avoidant coping partially mediates the relationship between experiencing 4+ stressful life events and quality of life (physical health) during the pandemic. Fig 2B shows that avoidant coping partially mediates the relationship between experiencing 4+ stressful life events and quality of life (psychological health) during the pandemic. Standardized coefficients and standard errors are shown. *p<0.05; **p<0.01; ***p<0.01.

Further, as shown in Table 3, we investigated if each coping strategy potentially mediated the relationships between SLEs and QOL physical health. The final model revealed that only avoidant coping is a potential partial mediator in the relationships between the QOL physical health dimension and experiencing 2, 3, and 4+ SLEs, with reductions in the SLE coefficients with the inclusion of avoidant coping. Fig 2A illustrates the partial mediation model for the relationship between experiencing 4+ SLEs, avoidant coping, and QOL physical health. Sensitivity analysis through Sobel tests confirmed that avoidant coping partially mediated the relationships between experiencing 2, 3, and 4+ SLEs and QOL physical health.

Table 4 shows the staged linear regression models for the relationships between SLEs and the QOL psychological health dimension. In the final model, both problem-focused coping (β = 0.57, σ = 0.12, *p* < 0.001) and emotion-focused coping (β = 0.86, σ = 0.13, *p* < 0.001) were positively associated with QOL psychological health, while avoidant coping (β = -1.33, σ = 0.12, *p* < 0.001) was negatively associated with QOL psychological health. Individuals who had an annual household income between $25,000- $34,999 (β = -5.54, σ = 2.42, *p* = 0.02) or $35,000-$49,999 (β = -4.88, σ = 2.05, *p* = 0.02), those who were cisgender female (β = -5.12, σ = 1.16, *p* = <0.001) or had an “other” gender identity (β = -10.02, σ = 3.87, *p* = 0.01), those who were divorced (β = -4.41, σ = 1.99, *p* = 0.03), in a relationship and cohabiting (β = -5.25, σ = 2.16, *p* = 0.02), in a relationship and not cohabiting (β = -6.41, σ = 2.56, *p* = 0.01), or single/never married (β = -5.74, σ = 1.49, *p*<0.001), and those who had a chronic physical health condition (β = -2.32, σ = 1.18, *p* = 0.049) or mental health condition (β = -9.69, σ = 1.18, *p* <0.001) had significantly lower QOL psychological health. On the other hand, increasing age had a small, positive association (β = 0.21, σ = 0.04, *p*<0.001) with QOL psychological health. Further, in the final model, experiencing 2 SLEs (β = -8.87, σ = 1.66, *p*<0.001) predicted the highest reduction in the coefficient for QOL psychological health, followed by experiencing 3 SLEs (β = -7.72, σ = 1.90, *p*<0.001), 4+ SLEs (β = -6.60, σ = 1.80, *p*<0.001), and 1 SLE (β = -3.52, σ = 1.42, *p* = 0.01).

Again, we tested potential mediation by each coping strategy in the relationships between SLEs and QOL psychological health and found that avoidant coping partially mediated these relationships, with reductions in the SLE coefficients. The partial mediation model for the relationship between experiencing 4+ SLEs, avoidant coping, and QOL psychological health is shown in Fig 2B. Sobel test findings also indicated that avoidant coping partially mediated the relationships between experiencing 1, 2, 3, and 4+ SLEs and QOL psychological health.

## Discussion

This study contributes to the emerging literature on the psychological impacts of the COVID-19 pandemic by shedding light on individuals’ experiences of SLEs, utilization of various coping strategies, and current QOL during this traumatic global event. Our findings indicate that the most common SLEs experienced during the pandemic were a decrease in financial status, personal injury or illness, and change in living conditions. We also found that, on average, respondents reported higher levels of problem-focused coping, followed by emotion-focused coping and avoidant coping. Further, our findings support our hypothesis that problem-focused coping and emotion-focused coping were significantly related to higher levels of QOL, whereas avoidant coping was associated with lower QOL. Importantly, consistent with our hypothesis, our study revealed that avoidant coping partially mediated the relationship between experiencing SLEs and lower physical and psychological QOL.

Studies have reported conflicting findings regarding the most prevalent SLEs experienced by individuals during the pandemic. A U.S.-based qualitative study by Jean-Baptiste et al. [45] found that the death of a loved one was the most common stressor experienced by respondents, followed by racism, discrimination including implicit bias and stereotyping, financial hardship and economic crisis, and personal health issues. On the other hand, a cross-sectional study conducted [46] in Iran found that the most prevalent stressor was the rise in essential good prices and that personal illness (i.e., being diagnosed with COVID-19) and the death of a loved one were ranked on the bottom of the list. Overall, our finding that experiencing financial difficulties and personal injury or illness were the most commonly experienced SLEs during the pandemic is consistent with this extant research.

Our findings corroborate existing literature indicating the positive association between the use of problem-focused coping and QOL pre-pandemic [47–49] and during the pandemic [28,34,35]. On the other hand, previous research, most of which was conducted pre-pandemic, has demonstrated inconsistent findings regarding the relationship between emotion-focused coping and QOL, with many studies pointing to a negative association between these two constructs [47,49,50]. A study conducted during the pandemic by Shamblaw et al. [28], however, has suggested that some emotion-focused coping strategies, such as the use of emotional support, may be related to well-being. As the pandemic instigated or exacerbated a wide range of unexpected and unpredictable stressors, such as personal illness, illness and deaths of loved ones, and unemployment, we posit that the use of emotion-focused coping was likely helpful in navigating these situations. Further, our findings regarding the inverse link between the use of avoidant coping strategies and QOL is supported by most pre-pandemic and pandemic-related literature [15,28,47,51,52].

Importantly, our study found that avoidant coping mediated the relationship between SLEs and lower QOL during the pandemic. Previous research has revealed similar findings in other contexts. Merluzzi et al. [15] discovered that the avoidant coping strategy of disengagement/denial coping mediated the associations between SLEs and emotion QOL and physical/functional QOL among cancer patients. Similarly, in a study by Langford et al. [31], disengagement coping mediated the association between SLEs and cancer-related distress. Further, Minahan et al. [32] found that avoidant coping mediated the relationships between pandemic-related stress and psychological outcomes, including depression, anxiety, and loneliness. However, to our knowledge, our study is the first to indicate that avoidant coping partially mediates the relationship between SLEs and QOL during the pandemic.

Our study’s findings have important clinical and public health implications. Greater exposure to stressors was linked with avoidant coping strategies, which were, in turn, associated with significantly lower QOL. Therefore, it is essential that mental healthcare and primary care providers dissuade the use of avoidant coping among patients, particularly those who experience elevated levels of stress. Alternatively, clinicians should promote the use of problem-focused coping, in general, to decrease the severity of stressors and the use of emotion-focused coping strategies when addressing uncontrollable or unpredictable stressors, such as large-scale traumatic events. Our study also highlights the most prevalent SLEs experienced during the pandemic. Hence, these findings call for public health and clinical interventions to address the long-term impacts of these stressors post-pandemic, especially among vulnerable groups such as racial/ethnic and gender minorities, cisgender women, lower-income individuals, unmarried individuals, and those with a chronic physical health condition, mental health condition, or disability.

Our study has some limitations that should be considered when interpreting its findings. First, as the survey was conducted in August 2021 and inquired about events that occurred over the time period of 17 months (since the lockdown in March 2020), responses may have been vulnerable to recall bias. Second, as this study was cross-sectional, causality cannot be assumed in the relationships between SLEs, coping strategies, and QOL, and mediation analysis is not ideal when the temporal relationships between these variables have not been established. However, though respondents were asked to report on SLEs experienced and coping strategies used during the pandemic, responses regarding QOL inquired about current (i.e., in the past month) perceptions and feelings. Third, we acknowledge that our observations about SLEs, coping strategies, and QOL experienced during the pandemic are not necessarily specific to the pandemic; for example, financial stressors are likely a prevailing stressor for many individuals and that avoidant coping is likely associated with the experience of more stressful life events and lower QOL, regardless of the pandemic. However, we contend that the pandemic provided an opportunity to examine these trends during a period of heightened global stress, and we were also able to discern the stressors that were likely more prevalent during the pandemic, such as personal illness and change in living conditions. Fourth, though *Prolific* creates nationally representative samples based on age, sex, and ethnicity data from the U.S. Census Bureau, our sample was not representative of the U.S. population in terms of ethnicity, thereby partially limiting the generalization of our findings to the U.S. population.

Despite these potential limitations, our study has critical implications for future research directions. Longitudinal research is needed to explore temporal relationships between the previous experience of SLEs, subsequent coping strategies that are employed, and current QOL. Further, as previous research has suggested that social support may act as a moderator in the relationship between stressors and QOL [53,54], researchers should consider the roles of social support and sense of community in the relationships between SLEs, coping strategies, and QOL, including the social relationships dimension of QOL, during global stressors such as the COVID-19 pandemic. Along these lines, traumatic events have been shown to lead not only to stress but also to posttraumatic growth [55]. Therefore, future research should explore the development of both posttraumatic stress and growth after the pandemic, as well as their relationships with coping strategies and QOL.

## Conclusion

Our study contributes to the literature by being the first, to our knowledge, to indicate that avoidant coping mediated the relationship between experiencing SLEs and lower physical and psychological QOL during the pandemic. Along these lines, we found that problem-focused coping and emotion-focused coping during the pandemic were significantly related to higher levels of current QOL, whereas avoidant coping was associated with lower QOL. Further, the most common SLEs experienced during the pandemic were a decrease in financial status, personal injury or illness, and change in living conditions. Our findings inform clinical interventions to help individuals adopt healthy behaviors to effectively manage stressors, especially large-scale traumatic events like the pandemic. Our study also sheds light on the most prevalent SLEs experienced during the pandemic, therefore calling for public health and clinical interventions to address the long-term impacts of these stressors post-pandemic, especially among vulnerable groups.
